# Lost Connection: A Case Report of Interrupted Pituitary Stalk Syndrome

**DOI:** 10.7759/cureus.60232

**Published:** 2024-05-13

**Authors:** Rana Bilal Idrees, Mariam Malik, Faisal Ehsan Cheema, Maham Khalid, Farwa Malik, Muhammad Hamid Chaudhary

**Affiliations:** 1 Radiology, INMOL Cancer Hospital, Lahore, PAK; 2 Radiology, Atomic Energy Cancer Hospital, Nuclear Medicine Oncology and Radiotherapy Institute (NORI), Islamabad, PAK; 3 Diagnostic Radiology, INMOL Cancer Hospital, Lahore, PAK; 4 Cardiac Surgery, Chaudhry Pervaiz Elahi Institute of Cardiology, Multan, PAK

**Keywords:** all neurology, clinical endocrinology, hypothalamic-pitutary-adrenal, mri imaging, neuro radiology

## Abstract

Pituitary stalk interruption syndrome is a triad of thin (<1 mm) or complete absence of the pituitary stalk with either an aplastic or ectopic posterior lobe of the pituitary gland and a hypoplastic or absent anterior lobe of the pituitary. Patients present with growth retardation, short height, seizures, intellectual disability, and absence of sexual maturation at the expected time. Here, we presented a case of a 12-year-old male with stunted growth. Upon examination, there was reduced height, more than 3 standard deviations below the average for his chronological age. Laboratory results showed reduced levels of growth hormone and thyrotropin. Dual-energy X-ray absorptiometry revealed osteoporosis, while an X-ray of the wrist for bone age corresponded to seven years. MRI imaging confirmed the classical triad of findings for pituitary stalk interruption syndrome. Consequently, the patient was referred back to the endocrinology clinic for further management.

## Introduction

Pituitary stalk interruption syndrome is a disorder in which there is a triad of a thin (<1 mm) or complete absence of the pituitary stalk with either an aplastic or ectopic posterior lobe of the pituitary gland and a hypoplastic or absent anterior lobe of the pituitary [1]. The incidence is rarely reported as 0.5 in every 100,000 live births [2], with a male predominance proposing X-linked inheritance [3]. The exact etiology is uncertain; there is thought to be an association with breech presentation, difficult or prolonged labor, forceps delivery, or birth trauma [4]. More recent studies have, however, proposed molecular defects in genes concerned with the development of the pituitary to be the cause of this syndrome [5].

The patient presentation is variable with regards to clinical, biochemical, and imagining workup; however, the most common features are growth retardation, short height, seizures, intellectual disability, and absence of sexual maturation at the expected time [6]. The severity of hormone deficiency influences the age of presentation, with patients either showing signs of deficient anterior pituitary hormones in infancy or childhood or panhypopituitarism in adults [2]. Growth hormone insufficiency is present in almost all patients and can be seen in isolation or combination with a deficiency of other anterior pituitary hormones. The function of the posterior pituitary usually remains normal, but in the case of aplasia or ectopia, dysfunction of the posterior pituitary may also be evident [7]. Advances in magnetic resonance imaging (MRI) have allowed for the diagnosis of this rare entity to be confirmed on cross-sectional imaging.

## Case presentation

**Figure 1:**
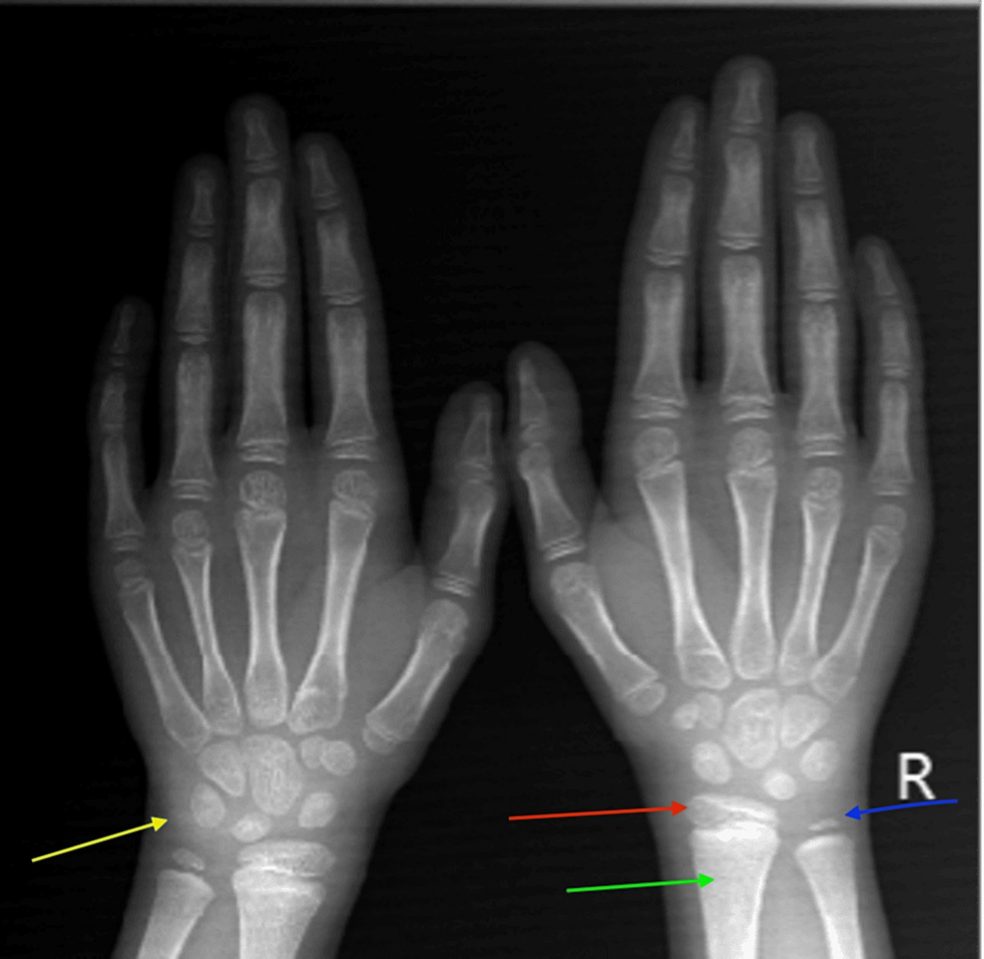
Ossification center of pisiform is absent (yellow arrow). The ulnar epiphysis (blue arrow) is present, and the width of the distal radial epiphysis (red arrow) exceeds the width of the visualized distal metaphysis (green arrow), correlating with a bone age of seven years according to the wrist radiograph.

Our patient, a 12-year-old male, was brought to the endocrinology clinic by concerned parents due to short stature. Upon examination, his height measured 115 cm (more than 3 standard deviations below the average for his chronological age), and his weight was 30 kg. Biochemical workup showed reduced growth hormone levels of 1.5 ng/mL and thyrotropin levels of 0.4 ng/mL. The bone age, as determined by the wrist radiograph, was seven years (Figure 1). Dual-energy absorptiometry for bone mineral density showed osteoporosis with a Z-score of -2.8 in the left femoral head and -2.4 in the L5 vertebral body. Contrast-enhanced MRI with the pituitary protocol was advised for further evaluation. The scan protocol was set with a 3 mm slice thickness and a field of view (FOV) of 15 cm. Sequences obtained included sagittal T2, coronal T2, axial non-contrast T1, dynamic post-contrast coronal T1, and post-contrast sagittal T1. The amount of intravenous Gadolinium administered was 3 mmol (according to 0.1 mmol/kg body weight).

**Figure 2:**
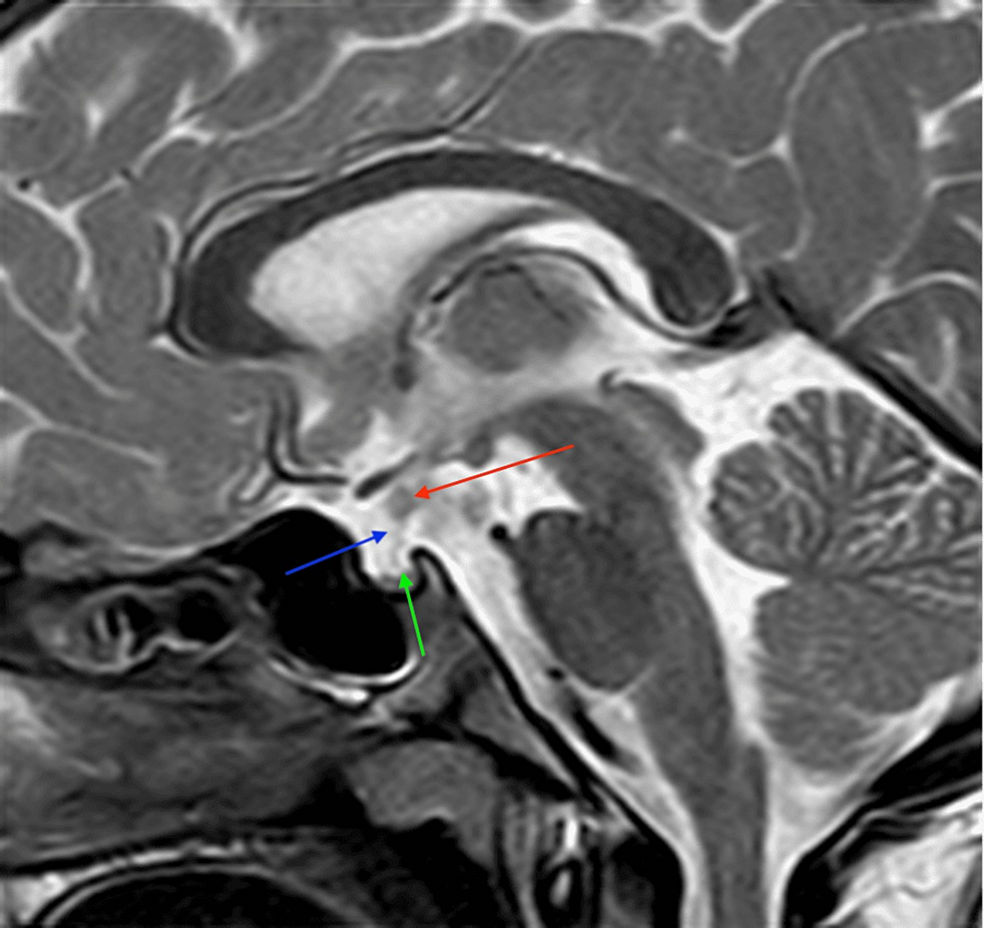
A thin-slice sagittal T2-weighted image (T2WI) at the level of the pituitary fossa reveals the classic triad of findings indicative of pituitary stalk interruption syndrome: an ectopically placed neurohypophysis (red arrow), a thin stalk measuring less than 1 mm (blue arrow), and a hypoplastic adenohypophysis (green arrow).

**Figure 3:**
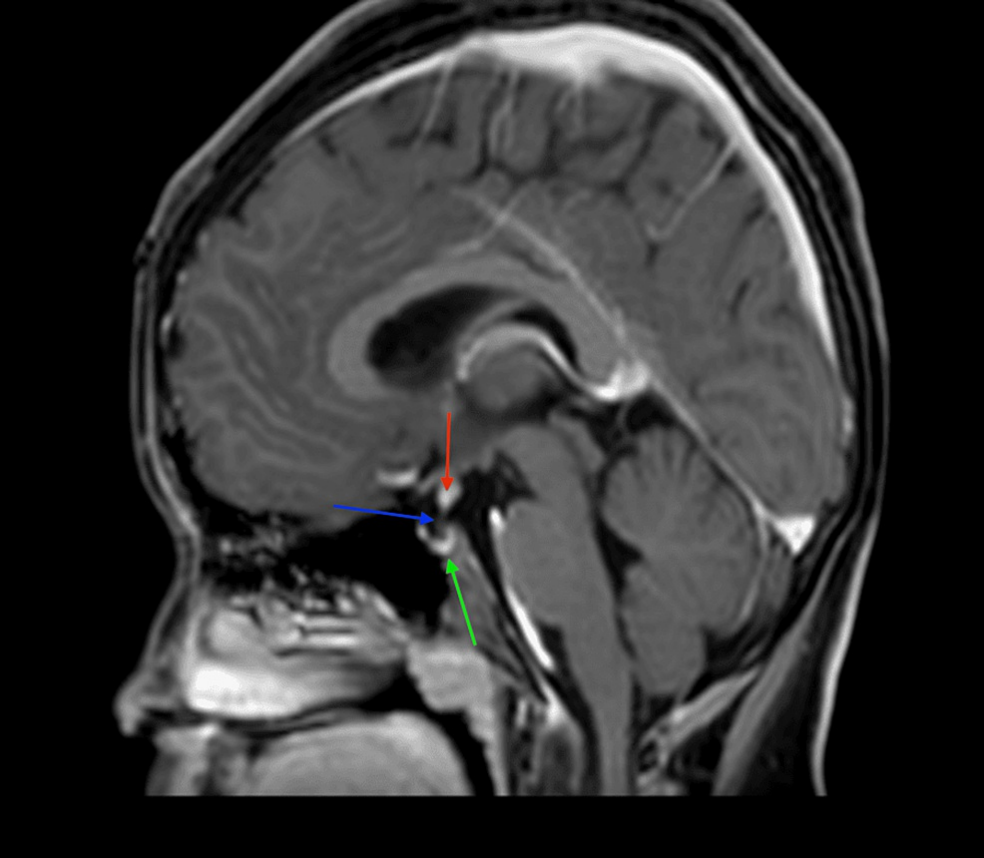
A post-contrast sagittal T1-weighted image (T1WI) at the level of the pituitary fossa reveals the classic triad of findings indicative of pituitary stalk interruption syndrome: an ectopically placed neurohypophysis (red arrow), a thin stalk measuring less than 1 mm (blue arrow), and a hypoplastic adenohypophysis (green arrow).

**Figure 4:**
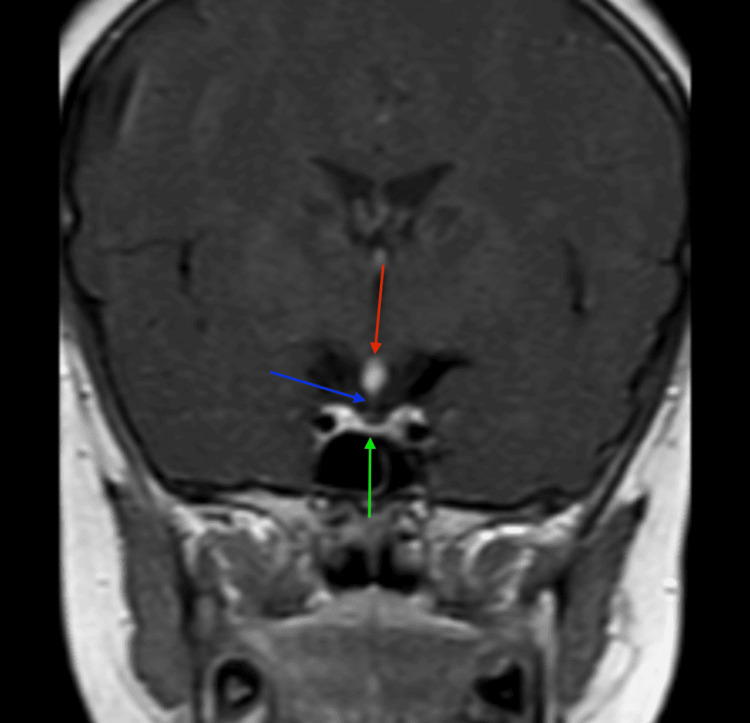
A post-contrast coronal T1-weighted image (T1WI) at the level of the pituitary fossa illustrates the classic triad of findings of pituitary stalk interruption syndrome, including the ectopically placed neurohypophysis (red arrow), thin stalk measuring less than 1 mm (blue arrow), and hypoplastic adenohypophysis (green arrow).

**Figure 5:**
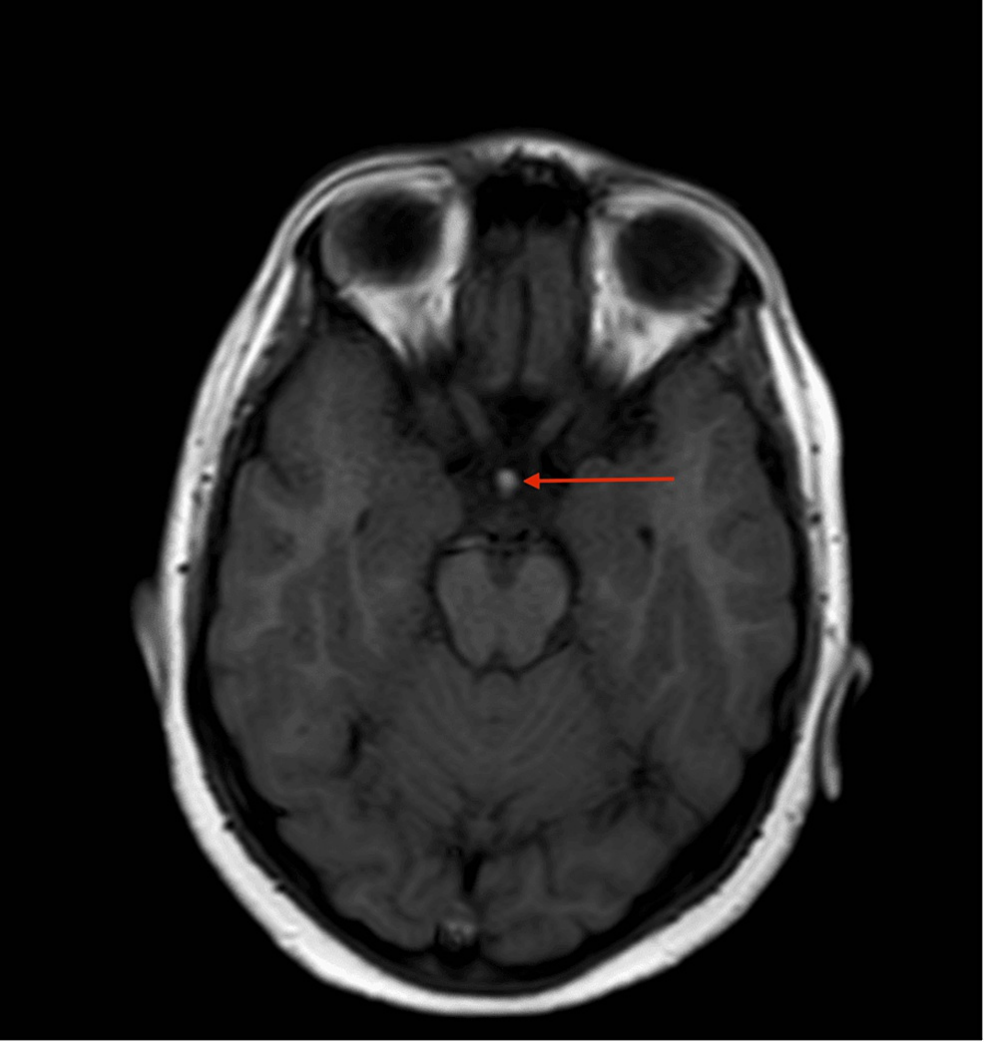
A non-contrast axial T1-weighted image (T1WI) at the level of the hypothalamus shows an abnormal nodular hyperintense focus (red arrow) at the level of the median eminence of the hypothalamus, representing an ectopically sited neurohypophysis.

The MRI showed hypoplastic adenohypophysis within the pituitary fossa (Figures 2-4). There was an ectopic positioning of the neurohypophysis situated within the median eminence of the hypothalamus, along with thinning of the pituitary stalk measuring less than 1 mm on sagittal and coronal images, confirming the triad of findings for interrupted pituitary stalk syndrome (Figures 2-5). Optic chiasm and bilateral cavernous sinuses were unremarkable (not shown in figures). 

The patient was referred back to endocrinology for growth hormone and thyrotropin replacement treatments.

## Discussion

In patients with pituitary stalk interruption syndrome, 100% suffer from short stature due to deficient growth hormone, 97.2% exhibit gonadotropin deficiency, 88.2% have reduced corticotropin levels, 70.3% show a reduction in thyrotropin, and 36.4% experience hyperprolactinemia [8,9]. A combination of three or more hormone deficiencies of the anterior pituitary was observed in 92.7% of patients [10]. The clinical features vary with the age of patients with prolonged neonatal jaundice, neonatal hypoglycemia, micropenis, and undescended testes in the newborns. In children, growth retardation is seen commonly, whereas in adolescence and early adulthood, delayed puberty is the most common presenting complaint.

Extra pituitary abnormalities are also seen with increased prevalence, including ophthalmologic, cardiac, cerebral, skin, and musculoskeletal defects [11]. There is a greater risk of congenital malformations in patients with isolated growth hormone deficiency when compared to patients with multiple anterior pituitary hormone insufficiency [12]. The ectopic posterior pituitary is present in the infundibular recess in 60.4% of the cases, while in 18.9% of the cases, it is seen in the hypothalamus [12], and this has a significant effect on prognostic outcomes as anterior pituitary hormonal deficiencies are more likely when the neurohypophysis is located in the median eminence of hypothalamus.

The diagnosis of this syndrome is based on clinical evaluation, hormonal workup, and contrast-enhanced MRI of the pituitary gland. Treatment includes life-long hormonal replacement therapies. The condition is associated with high morbidity and mortality if left untreated. In a meta-analysis by Pappachan et al., the age-specific standardized mortality ratio (SMR) was 2.92 (2.25-3.72) for childhood-onset hypopituitarism quoted with a 95% confidence interval [13].

The prognosis is based on early identification and initiation of treatment. Hence, close surveillance of growth in childhood increases the detection rate of growth abnormalities, enabling early identification of the cause and timely initiation of treatment. Once a diagnosis is established, it is essential to regularly monitor the patient for multiple pituitary hormone deficiencies with close follow-up.

## Conclusions

Because of the high morbidity and mortality, early detection and commencement of treatment are crucial in patients with pituitary stalk interruption syndrome. Hence, it is essential to keep this syndrome in differentials in patients with growth retardation. Early identification and the start of treatment before the fusion of epiphysis in children prevent short stature and increase the chances of attaining normal height.
